# Data Interworking Model and Analysis for Harmonization of Smart Metering Protocols in IoT-Based AMI System

**DOI:** 10.3390/s23062903

**Published:** 2023-03-07

**Authors:** Nogil Myoung, Yoojin Kwon, Myunghye Park, Changsoo Eun

**Affiliations:** 1 Digital Solution Laboratory, Korea Electric Power Corporation (KEPCO) Research Institute, 105 Munji-ro, Yuseong-gu, Daejeon 34056, Republic of Korea; 2 Department of Radio and Information Communication Engineering, Chungnam National University, 99 Daehak-ro, Yuseong-gu, Daejeon 34134, Republic of Korea

**Keywords:** smart metering, metering protocol, IoT protocol, smart meter gateway (SMGW), data concentrator unit (DCU), security

## Abstract

In the energy sector, since the adoption of remote device management for massive advanced metering infrastructure (AMI) devices and Internet of Things (IoT) technology using a representational state transfer (RESTful) architecture, a blurred boundary has been developed between traditional AMI and IoT. With respect to smart meters, the standard-based smart metering protocol, called the device language message specification (DLMS) protocol, still has a predominant role in the AMI industry. Thus, we aim to propose a novel data interworking model in this article that embraces the DLMS protocol in AMI using the most promising IoT protocol, the so-called lightweight machine-to-machine (LwM2M) protocol. We provide a 1:1 conversion model using the correlation of the two protocols with an analysis of the object modeling and resource management methods of both the LwM2M and DLMS protocols. The proposed model utilizes a complete RESTful architecture, which is the most beneficial in the LwM2M protocol. It improves the average packet transmission efficiency and packet delay on the plaintext and encrypted text (session establishment and authenticated encryption) by 52.9%p and 9.9%p, respectively, and by 11.86 ms for both cases, compared to the encapsulation method of the LwM2M protocol, KEPCO’s current approach. This work provides the key idea to unify the protocol for the remote metering and device management of field devices into the LwM2M protocol, and it is expected that this work will improve the efficiency in the operation and management of KEPCO’s AMI system.

## 1. Introduction

Renewable energy sources have been expanded dramatically with a large amount of solar and wind turbines in preparation for global climate commitments and meeting the goal of a nationally determined contribution target. Under this expansion, major utility companies have adopted advanced metering infrastructure (AMI) to offer remote metering and behind-the-meter services [1,2,3,4,5]. These services play a critical role in integrating intermittent and variable renewable energy sources into the power grid without any concern [6]. AMI plays the role of a supervisory control and data acquisition system or an advanced distribution management system (ADMS) for low-voltage power lines.

On the other hand, AMI has a unique characteristic in that it is the only system in the power grid that provides a two-way edge as a connection point, between consumers and utility. Hence, AMI enables real-time demand response services using dynamic tariffs such as time of use, critical peak pricing, and real-time pricing [7,8]. 

AMI devices consist of a head-end (HE), an aggregator, a communication network, and a smart meter. Due to the acceleration of AMI deployment, it is necessary to manage efficiently a large amount of AMI devices [9,10,11]. Thus, AMI devices, except smart meters, tend to adopt IoT protocols, which are representational state transfer (RESTful)-based [12,13] LwM2M or message queuing telemetry transport (MQTT) protocols for efficient device management such as parameter setting, initial authentication, provisioning, and firmware update.

However, it is difficult to apply these IoT protocols at smart meters since there is a strong policy to use metering protocols such as IEC 62056 series (DLMS) or ANSI C12.19/22 standards [14,15]. Therefore, these standard-based metering protocols do not switch to the IoT protocols.

Thus, in this article, we propose a data interworking model for the protocol conversion between the smart meters and AMI head-end system. This work is the novel attempt at proposing a conversion model, the so-called data interworking model, for the harmonization of IoT protocols and smart metering protocols. 

The main contributions of our work are summarized as follows: first, a data interworking model is proposed by analyzing and comparing the similarity of resource management between the DLMS and LwM2M protocols. It would efficiently collect the metering data and reduce the data traffic on the network. Second, we demonstrate the packet transmission efficiency and packet delay of the proposed model through the simulation of packet overhead.

The rest of this paper is organized as follows: Section 2 presents the review on the existing and recent work in relation to an IoT-based AMI head-end system and protocol conversion methods in the power grid. In Section 3, KEPCO’s AMI architecture and the plan for AMI enhancements are introduced. Section 4 covers the characteristics of the object modeling and resource management of both the DLMS and LwM2M protocols, and then a novel data interworking model is proposed. In Section 5, we analyze the packet transmission efficiency and packet delay of the proposed data interworking model. In the final section, we conclude our work.

## 2. Related Works

As described in Section 1, the IoT protocols have been widely investigated academically for power grids [16,17,18] and recently adopted in AMI [19,20]. Lioret et al. [18] proposed an integrated IoT architecture for smart meter network to be deployed in smart cities. They discuss the communication protocols, data format, data gathering procedure, and decision system based on big data. Orlando et al. [21] suggested a low-cost smart meter architecture for AMI and made a prototype of smart meters that follow the basic IoT principles for smart grid management. Furthermore, they verify the performance of the smart meters using a hardware-based digital real-time simulator. Chen et al. [22] proposed a smart metering architecture for IoT-based smart meters. They design it using an edge computing concept to overcome the latency and bandwidth problems. Yoon et al. [23] proved that smart meters can perform remote metering using the RESTful application programming interface at the AMI head-end system and the implementation of the MQTT protocol and hypertext transfer protocol (HTTP).

There are a few previous studies focused on ensuring a data interworking between heterogeneous protocols in the power grid. Zhang et al. [24] suggested a conversion model between the Modbus protocol, one of the most popular protocols in the control system, and the IEC 61850 standard-based substation automation protocol. There is also a naive approach using a mapping table with the conversion method between the Modbus protocol and distributed network protocol [25]. However, the mapping table approach leads to not only a burden with manual table setting and management, but also difficulty in updating policy flexibly in circumstances of remote metering or configuration change that may occur. Myoung et al. [26] suggested a data interworking concept for the first time in AMI to secure interoperability between the DLMS and LwM2M protocols without using the mapping table.

## 3. KEPCO’s AMI Architecture and Enhancement Plan

The long-term roadmap of KEPCO’s AMI has been established according to the intelligent power grid roadmap plan of the Korean government in 2010 [27]. In 2013, the AMI project was started, with AMI being installed for 2 million customers, which will expand to 22.5 million customers by 2025, with an investment of approximately USD 15 billion [9,10]. By the end of 2022, AMI had been installed for 12.5 million customers, providing basic services such as remote metering, outage management system (OMS), fraud detection, and secondary monitoring of distribution service transformers. 

### 3.1. AMI Architecture in KEPCO

**Figure 1:**
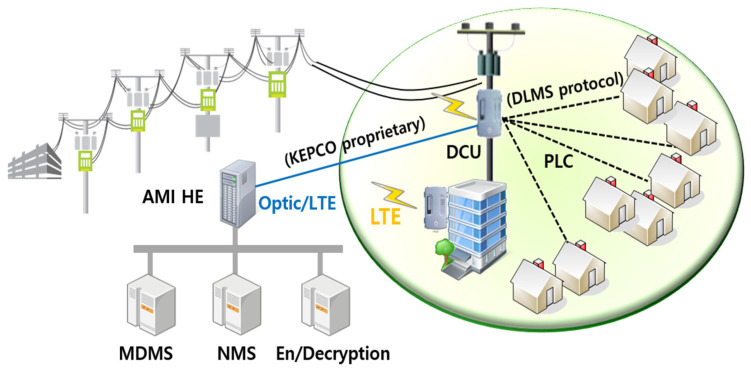
KEPCO AMI architecture.

Figure 1 depicts KEPCO’s AMI architecture. KEPCO, the only one utility company in Korea, selects a data concentrator unit (DCU) type to provide an aggregator role as well as a protocol conversion feature, similar to other utility companies with AMI deployment [5,7,8,28]. Private optic communication and the IEC 12139-1 (high-speed power line communication) standard [29] are mainly used accordingly in DCUs as a back-haul and neighborhood area network. The DCU is installed near the distribution service transformer. 

This DCU measures the voltage and current values of the secondary distribution service transformer. Additionally, it provides the overload monitoring and fraud detection capability of the distribution service transformer [30]. The lightweight version of DCUs installed beside the merging unit of multiplex housing does not have those functionalities. It uses long-term evolution (LTE) as a back-haul, leased from telecom service providers.

**Figure 2:**
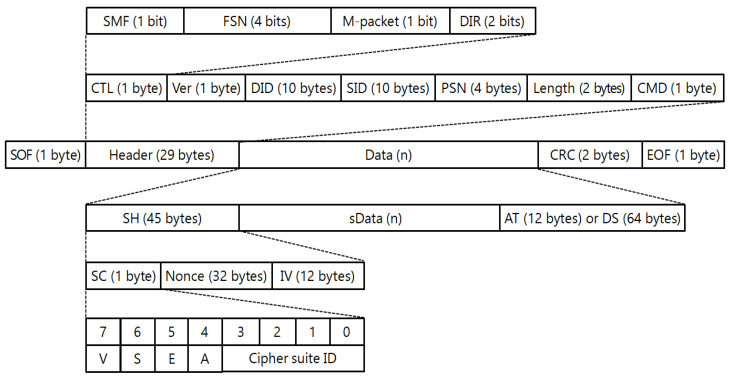
Protocol structure between data concentrator unit and AMI head-end system.

The remote metering requirement of the DCU is defined as follows: two hundred smart meters at maximum with pre-defined metering items are collected every fifteen minutes using the DLMS protocol [31], and they are sent to the AMI head-end system using KEPCO’s proprietary protocol. Figure 2 shows the packet structure of KEPCO’s proprietary protocol used between the DCU and AMI head-end system [10]. 

The structure of a packet consists of the start of frame (SOF), header, data, cyclic redundancy check (CRC), and end of frame (EOF) fields. The header field consists of control (CTL), destination ID (DID), source ID (SID), and command (CMD). It is distinguished by the request and response on remote metering depending on the direction (DIR) of the control field. The data field consists of a security header (SH) for cyber security requirements and its corresponding authentication tag (AT) or digital signature (DS) field. In addition, the security header consists of the security control (SC), nonce, and initial vector (IV) fields. It designates the cipher suite ID, digital signature generation (S) and verification (V), encryption (E), and authentication (A) using the security control field. Lastly, the metering information is defined in the data field with an array structure of meter ID, meter type, measurement type, and measurement values in order.

### 3.2. AMI Enhancements in KEPCO

KEPCO’s AMI architecture and its application services were originally designed to provide an automatic meter reading (AMR) service almost one decade ago. Additional behind-the-meter services for AMI, similar to the control of smart inverters, as well as efficient device management services for AMI field devices, are in high demand since AMI has been deployed for more than 12.5 million customers at a site [10].

Moreover, AMI head-end system to smart meter as an end-to-end security requirement is introduced to enhance cyber security and protect against the increasing attack surface due to the nationwide AMI deployment [10,32]. In Table 1, a comparison of the major characteristics between KEPCO AMI 1.0 and 2.0 is summarized.

#### 3.2.1. Smart Meter Gateway (SMGW) and Security Enhancement

The core device of the AMI system, the DCU, has been upgraded to an SMGW for multiple services. The SMGW runs on a software platform which embeds various new applications besides the remote metering service [10]. Specifically, the SMGW adds the functionality to expand the hosting capacity of renewable energy sources and prevent the distribution line from overload and overvoltage issues through the integration with an ADMS. Therefore, it performs power control over renewable energy sources and eventually provides behind-the-meter services. 

To support end-to-end security, a secure element with a Korean cryptographic module validation program and public key infrastructure (PKI) is planned for adoption in smart meters in 2023 [10,32]. Table 2 shows DLMS security comparisons between the DCU/SMGW and smart meter for AMI 1.0 and 2.0. The only security applied between the DCU and smart meter in AMI 1.0 is password-based mutual authentication. 

In AMI 2.0, after completing digital certificate-based mutual authentication (mechanism ID 7) between the SMGW and smart meter, key agreement is performed based on a static unified model to generate a shared secret [10,33]. With the shared secret, every remote metering and parameter setting in a packet uses a service-specific dedicated ciphering application protocol data unit (APDU) for authenticated encryption. In addition, non-repudiation functionality with a general signing APDU is used only for billing information. 

For security between the SMGW and AMI 2.0 head-end system, the datagram transport layer security (DTLS) protocol is used [34] because the LwM2M protocol adopts the DTLS protocol for security. The only cipher suite is ECDH-ECDSA-AES-GCM-128-SHA-256 for all ranges of communication. KEPCO has defined its digital certificate based on X.509 [10]. 

#### 3.2.2. LwM2M Protocol Adoption and Limitations

The simple network management protocol (SNMP) is used for AMI device management in KEPCO [9]. However, it is originally developed for communication devices such as routers, thus this protocol is heavy and outdated. Moreover, it only provides a network status monitoring service.

Therefore, to replace it, the LwM2M protocol is chosen to provide lifecycle remote device management and remote metering services for massive AMI devices. It uses a RESTful architecture and HTTP methods (GET/POST/PUT/DELETE), which have the advantages of easy object modeling and resource management, good scalability, and compatibility [12,13]. Additionally, it uses the constrained application protocol (CoAP) as an application layer protocol to maximize the network throughput by simplifying the packet size. Mutual authentication, key agreement, and encryption are implemented using the DTLS protocol.

Since the LwM2M protocol is adopted between the AMI 2.0 head-end system and SMGW, KEPCO has unified the protocols for remote device management and remote metering into the LwM2M protocol. Meanwhile, to provide backward comparability with the existing proprietary protocol, KEPCO temporarily uses an object ID = 19 (BinaryAppDataContainer)-based encapsulation method defined in the LwM2M protocol [10,35]. 

This encapsulation method is unnecessarily implemented in the AMI 2.0 head-end system with both a proprietary protocol client and CoAP client, thus resulting in resource overhead. Ultimately, there is no need for the AMI 2.0 head-end system to develop a translator for the proprietary protocol, reduce the protocol overhead, and utilize a complete RESTful architecture, which is the most beneficial in the IoT protocol. 

## 4. Data Interworking Model for Remote Metering

In this article, we propose a data interworking model between the DLMS and LwM2M protocols as a substitute for the current encapsulation method of the LwM2M protocol. The proposed model uses an algorithm-based dynamic conversion method, not a static-based mapping table method.

The key to adopt smart metering protocols in an IoT-based AMI system is to minimize the role of DLMS protocol usage since it has the uniqueness of the protocol itself, compared to the IoT protocols. We may assume that the AMI modem should perform the protocol conversion task since it is the nearest to smart meters in the field. However, to consider the viewpoint of the practical operation, the SMGW should perform the protocol conversion task owing to its outstanding processing power, meter data aggregation capability, and gateway functionality.

### 4.1. Object Modeling and Resource Management for DLMS and LwM2M Protocols

In the past, the DLMS protocol operated on a server–client model to support the remote metering or parameter settings of smart meters. Recently, the DLMS protocol adopts a push operation to send real-time event messages [14,15]. 

**Figure 3:**
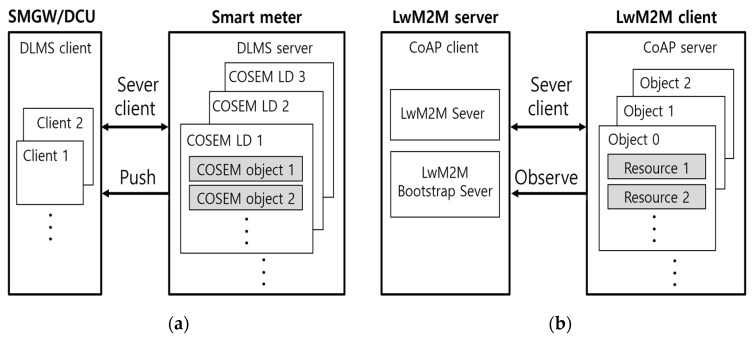
Service models. (**a**) DLMS service model; (**b**) LwM2M service model.

As described in Figure 3a, one smart meter can be logically modeled with multiple DLMS servers, and each server is called a companion specification for energy metering (COSEM) logical device. A COSEM logical device consists of various COSEM objects which represent the measurement of voltage, current, etc., and the metering data. Remote metering is a process where the DLMS client reads the COSEM object that has been modeled in the DLMS server. A smart meter holds at least dozens to thousands of COSEM objects and manages them accordingly. 

To ensure interoperability, every COSEM object is modeled using the COSEM interface class defined and managed in the DLMS user association [15,36]. Each COSEM interface class has one unique identification (ID), called the class ID, and is composed of multiple attributes and methods. The first attribute is always used for the object identification system (OBIS) code, and the rest of the attributes represent measurement, metering value, and its unit.

Table 3 describes the OBIS code with a hierarchical structure composed of six groups, each with a size of one byte. Group A is for the type of energy, such as electricity, gas, or water. Group B categorizes the measurement or communication channel. Group C is related to Group A to specify the physical data object such as current, voltage, volume, and temperature. Group D and Group E are defined to calculate or categorize the data in Group C. Lastly, Group F is for historical data using timing information. To summarize, the core concept of resource management in the DLMS protocol is the hierarchical structure of the COSEM object model that consists of the class ID, OBIS code, and attributes.

The LwM2M protocol operates on a server–client model, like the DLMS protocol. Additionally, it uses an observe method for real-time event messages. As shown in Figure 3b, the LwM2M device, which runs on a CoAP server, may have various logical LwM2M objects. The data value for the LwM2M object is represented by resources. To specify objects and resources, object instance and resource instance are used, respectively. The remote data collection process in the LwM2M protocol is similar to that of the DLMS protocol. It is a process where the CoAP client reads the resource value of the LwM2M object that has been modeled in the CoAP server. 

**Figure 4:**
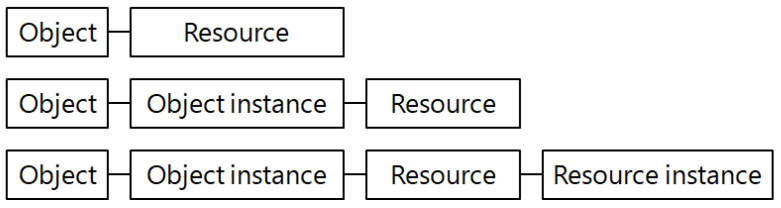
LwM2M URI structures.

Eventually, as described in Figure 4, the core concept of resource management in the LwM2M protocol is to combine the LwM2M objects and resources into a uniform resource identifier (URI) with a hierarchical structure. 

### 4.2. Proposed Data Interworking Model

The DLMS and LwM2M protocols have similar resource management features, as summarized in Table 4. We propose a 1:1 conversion model using the similarity of each parameter that is correlated between the DLMS and LwM2M protocols. The proposed model is designed in consideration of the scalability of remote integrated metering and the smart meter installation circumstances at a site. The proposed model should meet the following requirements:

- Support automated, mutual conversion functionality based on an algorithm;
- Be applicable to all energy sources—not only electricity, but also water, gas, etc.;
- Be an identifier in the case of a multi-complex house, with up to a maximum of 15 smart meters connected to 1 AMI modem.

**Figure 5:**
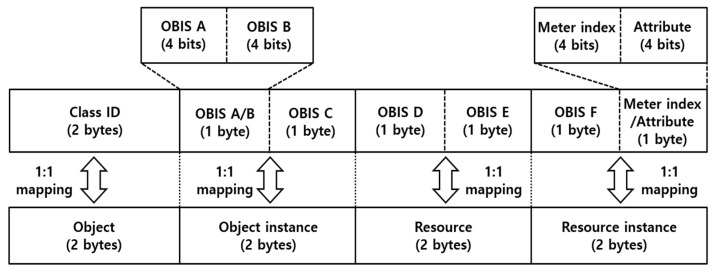
Resource conversion method between DLMS and LwM2M protocols.

The key structure for resource management in the DLMS protocol with three parameters, i.e., ‘class ID + OBIS code + attribute’, is converted 1:1 to the key structure for resource management in the LwM2M protocol with four parameters, i.e., ‘object + object instance + resource + resource instance’, in the URI structure, as shown in Figure 5. It is best to minimize the transmission size of the LwM2M packet by reducing the URI size itself. 

However, the LwM2M object and its instance have a maximum size of 2 bytes accordingly. For the 1:1 conversion to the resource management structure in the DLMS protocol, the URI structure consisting of four parameters, i.e., ‘object + object instance + resource + resource instance’, has to be used. 

Thus, each parameter is converted accordingly as follows: class ID is converted to object; OBIS code groups A/B/C are converted to object instances; OBIS code groups D/E are converted to resources; OBIS code group F/meter index/attribute are converted to resource instances. Since OBIS code group A has the value range of 0~15, a size of 4 bits is allocated to cover all energy sources. Since OBIS group B has the value range of 0~64, but uses a value of 0~1 in most cases, a size of 4 bits is allocated, as expressed in Table 3. The meter index allocates 4 bits of size for up to 16 smart meters.

### 4.3. Conversion Example with the Proposed Data Interworking Model

This section introduces an example of a load profile using the data interworking model between the DLMS and LwM2M protocols and its conversion algorithm, as shown in Figure 5 and Algorithm 1. The index of a smart meter is assumed to be 1. Detailed items of the DLMS protocol are used in KEPCO’s AMI. The load profile has 10 items, as shown in Table 5. 

Let us calculate the URI value of active power (+). The first field of URI, object, is calculated as ‘03’ following the conversion algorithm in steps 1~3. The second field of URI, object instance, is calculated as 17 (1 × 16 + 1) × 256 + 1 = ’4353’ following the conversion algorithm in steps 4~7. The third field of URI, resource, is calculated as 256 × 8 + 0 = ‘2048’ following the conversion algorithm in steps 8 ~ 10. For the final step, the last field of URI, resource instance, is calculated as 256 × 255 + 18 (16 × 1 + 2) = ‘65298’ following the conversion algorithm in steps 11~14. As a result, the conversion of active power (+) into /03/4353/2048/65298 is completed with the URI. Other load profile items are converted in the equivalent steps with active power (+) to create URI values.
**Algorithm 1** DLMS protocol to LwM2M protocol conversion algorithm1: DLMS client extracts the smart meter index, class ID, OBIS code, and attribute index from the received DLMS packet. 2: Confirm class ID to calculate the object value. 3: Calculate the first URI value with class ID (decimal). 4: To calculate an object instance, extract group A, B, and C from the OBIS code. 5: The upper byte of the object instance is calculated by group A (decimal) × 16 + group B (decimal). 6. The lower byte of the object instance is calculated by group C (decimal). 7: The second URI is calculated by the upper byte of the object instance (decimal) × 256 + the lower byte of the object instance (decimal). 8: To calculate a resource, extract group D and E from the OBIS code. 9: The upper and lower byte of the resource is calculated by group D (decimal) and group E (decimal). 10: The third URI is calculated by the upper byte of a resource (decimal) × 256 + the lower byte of a resource (decimal). 11: To calculate the resource instance, extract group F from the OBIS code, smart meter index, and attribute index. 12: The upper byte of a resource instance is calculated by group F (decimal). 13: The lower byte of a resource instance is calculated by smart meter index (decimal) × 16 + attribute index (decimal). 14: The fourth URI is calculated by the upper byte of the resource instance (decimal) × 256 + the lower byte of the resource instance (decimal). 15: Compose URI with object/object instance/resource/resource instance in order.

## 5. Analysis of the Packet Transmission Efficiency and Packet Delay for the Data Interworking Model with KEPCO’s AMI Cyber Security Policy

We compare the proposed model with an encapsulated method of the LwM2M protocol currently used by KEPCO on the packet transmission efficiency and packet delay in a quantitative advantage. In addition, we analyze the impact of these performance metrics according to the execution of KEPCO’s AMI 2.0 cyber security policy, as shown in Table 1 and Table 2.

**Figure 6:**
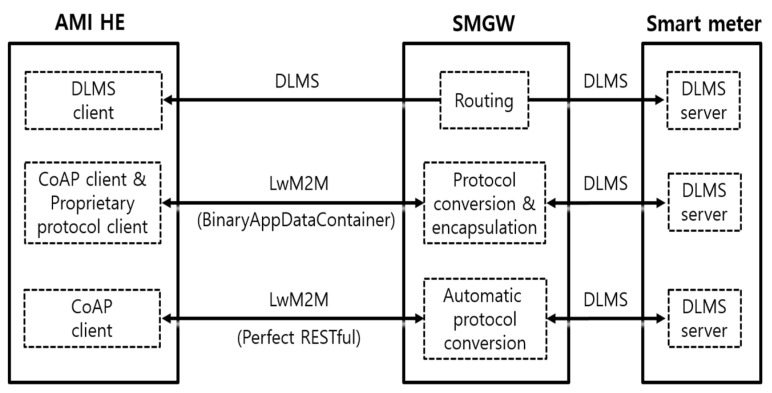
Three remote metering approaches to compare performance metrics.

Figure 6 summarizes the three different approaches of remote metering to compare the performance metrics. The first approach runs on the full DLMS TCP/IP profile from the AMI head-end system to smart meter, which is the baseline for the comparative analysis of the packet transmission and packet delay. Since the remote metering method used by each utility company and related protocols (standard, proprietary, or hybrid) are different, it is assumed that the first end-to-end standard-based approach is the state-of-the-art remote metering method. There is no protocol conversion task in the SMGW acting as a network router in this first approach. In the second approach, which is applied to the KEPCO AMI 2.0 project, the SMGW converts the DLMS protocol into the LwM2M protocol and sends it to the AMI 2.0 head-end system. However, to support interoperability with the existing proprietary protocol, KEPCO adopts an encapsulation method with ‘BinaryAppDataContainer’ defined in the LwM2M protocol [10,35]. In the last approach, the SMGW 1:1 converts the DLMS protocol into the LwM2M protocol and sends it to the AMI head-end system proposed in this article.

### 5.1. Analysis of the Packet Payload and Remote Metering Items

The most critical profile items for pricing, demand response, and behind-the-meter services are selected from various remote metering profiles KEPCO collects periodically [10]. Table 6 introduces the load profile, billing profile, maximum demand profile, instantaneous profile with each specific component, and the size of each payload. Most electric utility companies use profile methods in remote metering to minimize the number of transactions. Approximately, the size of the payload for each profile is usually dozens to hundreds of bytes. Table 7 depicts the required packet size for each of the three remote metering approaches. The following paragraph describes the calculation steps for the packet transmission size focused on the load profile.

The first approach requires 6 bytes of packet size for the DLMS header and 26 bytes of packet size for the body, i.e., 32 bytes for the request packet in total. For the response packet, it requires 67 bytes of packet size with 6 bytes of header and 61 bytes of body. The 47 bytes of packet size for the load profile increases to 61 bytes through the type–length–value (TLV) encoding in the DLMS protocol.

The second and third approaches use the CoAP protocol; thus, they both require 8 bytes of packet size for the CoAP header. Meanwhile, since the second approach with the encapsulation method uses 5 bytes of packet size for the CoAP option, (/19/2), it requires 13 bytes of packet size for the request packet, the minimum size for the request packet among the three different approaches. For the response packet, it requires 157 bytes of packet size, which is the maximum size among the three different approaches. This is because the 47 bytes of packet size for the load profile increases to 72 bytes through Base64 encoding, and an additional 77 bytes is required to represent the JavaScript object notation (JSON) header used in the KEPCO proprietary protocol.

The third approach requires 12 bytes more than the second approach due to the 17 bytes of packet size for the CoAP option, i.e., 25 bytes of request packet in total. Meanwhile, since the third approach does not require an encoding task for the load profile, it requires 55 bytes of packet size for the response packet, the minimum size for a response packet among the three different approaches. Based on the same calculation steps as for the load profile, the transmission packet size of the billing profile, maximum demand profile, and instantaneous profile can be calculated.

### 5.2. Analysis of the Security Overhead

According to KEPCO’s roadmap, end-to-end security is planned to be applied from 2024 [10]. Thus, it is crucial to understand the effect of the security overhead on the packet transmission and packet delay depending on three different approaches to remote metering. Since KEPCO has selected a PKI-based security policy, a digital certificate is used for authentication, key agreement, and authenticated encryption in the DLMS and DTLS protocols. Both protocols provide similar security features and procedures, but they differ in the required packet size [14,33,34].

Table 8 depicts the overhead packet size required in the DLMS and DTLS protocols for the adoption of the ECDH-ECDSA-AES-GCM-128-SHA-256 cipher suite using 472 bytes of the digital certificate defined by KEPCO. The DLMS protocol requires a total of 1639 bytes for the application association and static unified model-based key agreement procedure. Application association is used for mutual authentication based on digital certificates and extended DLMS (xDLMS) service conformance negotiations. The DTLS (version 1.2) protocol requires a total of 2364 bytes during the handshake phase.

**Figure 7:**
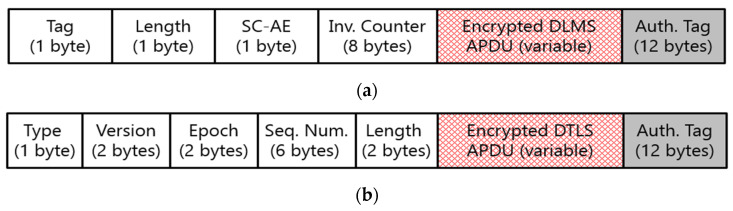
Ciphering APDU structure. (**a**) Structure of service-specific dedicated ciphering DLMS APDU [14]. (**b**) Structure of ciphering DTLS APDU [34].

Figure 7 shows the structure of the ciphering APDU used for authenticated encryption in the DLMS and DTLS protocols. These protocols require 23 bytes and 25 bytes of overhead size, respectively, to apply authenticated encryption. The fundamental remote metering process is initialized with the session establishment, and then the scheduled metering is performed and the session is released.

### 5.3. Analysis of the Performance Metrics

We use Equations (1) and (2) to analyze the packet transmission efficiency and packet delay of three different approaches of remote metering referred to in Figure 6, respectively. We narrow down the target of the analysis into the application layer, regardless of lower communication layers. The data rate of LTE Cat. M1, 300 kbps, is applied in Equation (2) because KEPCO uses it as the back-haul network. To specify the effect of the security overhead, the session establishment (DTLS handshake or DLMS application association with key agreement) overhead, authenticated encryption overhead, and total overhead are considered, respectively.

(1) $$\mathrm{Transmission}\;\mathrm{efficiency}\;\left(\%\right)=\frac{full\;DLMS\;packet\;size}{packet\;size\;of\;each\;approach}\times 100$$

(2) $$\mathrm{Packet}\;\mathrm{delay}\;\left(s\right)=\frac{packet\;size\;of\;each\;approach}{\mathrm{data}\;\mathrm{rate}\;\mathrm{of}\;\mathrm{LTE}\;\mathrm{Cat}.\;M1}$$

**Figure 8:**
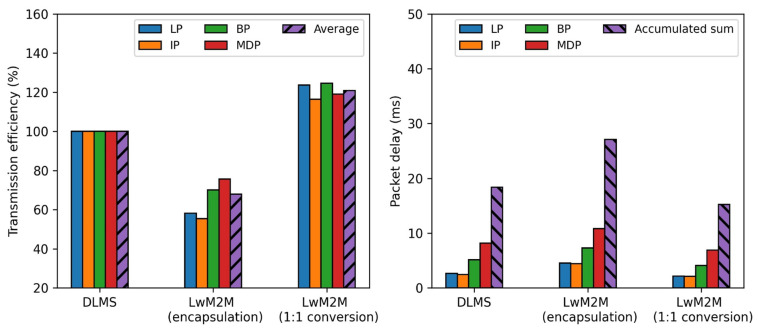
Comparison of the performance metrics (plaintext).

Figure 8 summarizes the performance metrics with only plaintext for the three different approaches to remote metering. It also presents arithmetic mean value (average) of the packet transmission efficiency and accumulated sum of the packet delay. The result shows that the proposed approach improves the packet transmission efficiency, on average, by 20.8%p and 52.9%p, respectively, and packet delay, in total, by 3.17 ms and 11.86 ms, respectively, compared to the full DLMS protocol approach and encapsulation approach of the LwM2M protocol. The proposed approach has much higher efficiency and lower delay than that of the encapsulation approach of the LwM2M protocol, since there is no additional JSON Base64 encoding on the payload.

**Figure 9:**
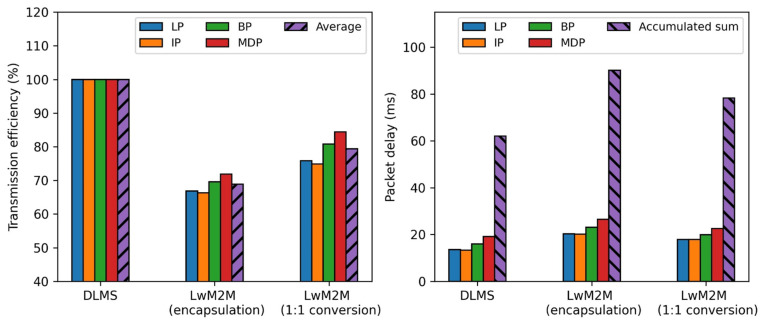
Comparison of the performance metrics (plaintext after session establishment).

Figure 9 summarizes the performance metrics with plaintext after the session establishment for the three different approaches to remote metering. The result shows that the proposed approach improves the packet transmission efficiency, on average, by 10.4%p and the packet delay, in total, by 11.86 ms, compared to the encapsulation approach of the LwM2M protocol, whereas it deteriorates the packet transmission efficiency, on average, by 20.6%p and the packet delay, in total, by 16.16 ms, compared to the full DLMS protocol approach. Both the encapsulation approach and proposed approach using the LwM2M protocol deteriorate the performance metrics, since the packet size of the DTLS protocol (2364 bytes) needed for the session establishment is relatively larger than that of the DLMS protocol (1639 bytes).

**Figure 10:**
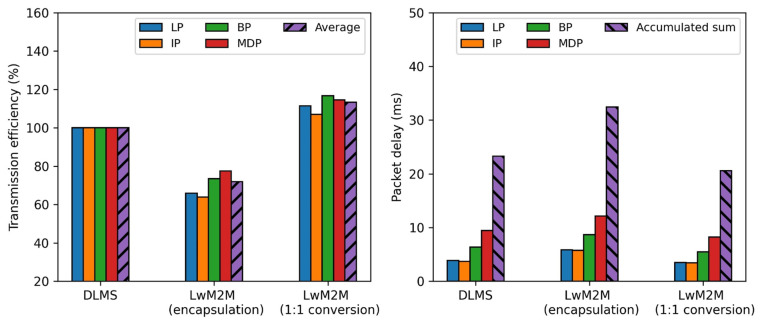
Comparison of the performance metrics (authenticated encryption).

Figure 10 summarizes the performance metrics of authenticated encryption without the session establishment for the three different approaches to remote metering. The result shows that the proposed approach improves the packet transmission efficiency, on average, by 13.3%p and 41.5%p, respectively, and packet delay, in total, by 2.74 ms and 11.86 ms, respectively, compared to the full DLMS protocol approach and encapsulation approach of the LwM2M protocol. The result using the DTLS protocol does not show the equivalent improvement, compared to the plaintext case, since the overhead packet size of the DTLS protocol (25 bytes) needed for authenticated encryption is larger than that of the DLMS protocol (23 bytes).

**Figure 11:**
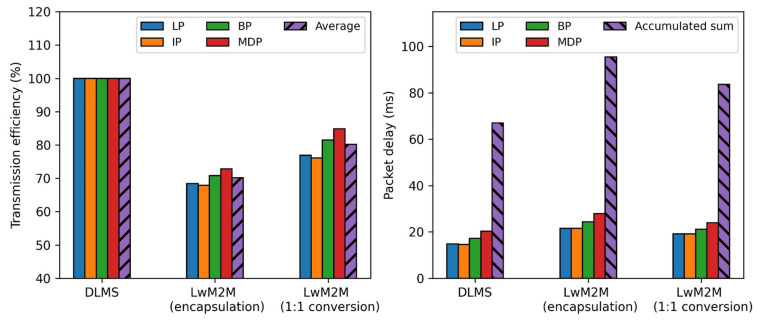
Comparison of the performance metrics (auth. encryption after session establishment).

Figure 11 summarizes the performance metrics of authenticated encryption with the session establishment for the three different approaches. The result shows that the proposed approach improves the packet transmission efficiency, on average, by 9.9%p and the packet delay, in total, by 11.86 ms, compared to the encapsulation approach of the LwM2M protocol, whereas it deteriorates the packet transmission efficiency, on average, by 19.8%p and the packet delay, in total, by 16.58 ms, compared to the full DLMS protocol approach.

To sum up, depending on whether cyber security is applied or not, the proposed approach improves the performance metrics, compared to the encapsulation approach of the LwM2M protocol. However, its performance metrics do not show the significant improvement, compared to the full DLMS approach, since we apply the session establishment to be performed each time for scheduled metering. Further, the overhead of the session establishment for the DTLS protocol is larger than that of the DLMS protocol. If the session establishment is adjusted into a longer period than the currently scheduled metering, every fifteen minutes, the performance of the proposed approach will be better because it does not require any encoding overhead for the payload.

When applying the ECDH-ECDSA-AES-GCM-128-SHA-256 cipher suite for PKI security, both in the DLMS and LwM2M protocols, we find that this cipher suite requires a relevantly large packet size during the session establishment phase in comparison with the payload size for transmission. It should be considered for any utility company’s AMI management policy to trade-off between periodic metering with session establishment and a smaller number of session establishments performed by compromising the risk of security. We use two performance metrics, the packet transmission efficiency and packet delay, to evaluate the proposed model. The relationship between the packet transmission efficiency and packet delay is an inverse because we use the fixed 300 kbps data rate and do not consider the characteristics of lower communication layers.

## 6. Conclusions

KEPCO has launched the AMI 2.0 project, which aims to improve AMI 1.0 with restricted remote metering functionality, into the adoption of behind-the-meter service support, cyber security functionality, and the efficient remote management capability of field devices. For these purposes, the most promising IoT protocol, the so-called LwM2M protocol, has been newly selected for the AMI 2.0 project.

However, the typical metering protocol, the so-called DLMS protocol, has been used for all metering data in AMI until today. This has introduced the problems of interoperability between two heterogeneous protocols and the exchange of data between them. Thus, we propose a new data interworking model that embraces the DLMS protocol in the AMI 2.0 head-end system using the LwM2M protocol.

First, the characteristics of the object modeling and resource management methods of both the LwM2M and DLMS protocols are analyzed. Second, a new 1:1 conversion model using the correlation of the two protocols is provided, and thorough conversion steps with an example of a load profile are presented. The proposed model (1) utilizes a complete RESTful architecture, which is the most beneficial in the LwM2M protocol, and (2) improves the packet transmission efficiency and packet delay, compared to the encapsulation method of the LwM2M protocol, KEPCO’s current approach. To compare the performance metrics quantitatively, we analyze the payload size of a packet with critical metering items used by KEPCO for three different approaches to remote metering. Finally, we apply the security overhead in a packet through the session establishment and authenticated encryption concerning the adoption of PKI-based cyber security.

The proposed methodology improves the average packet transmission efficiency and packet delay on the plaintext and encrypted text (session establishment and authenticated encryption) by 52.9%p and 9.9%p, respectively, and by 11.86 ms for both cases, compared to the encapsulation method of the LwM2M protocol. This work provides the key idea to unify the protocols for remote metering and device management of field devices into the LwM2M protocol. We believe that this work will improve the efficiency of the operation and management of KEPCO’s AMI system. Future work should include field tests to verify the applicability of the proposed algorithm and deployment of the data interworking model.

## Figures and Tables

**Table 1:** Function comparison between KEPCO AMI 1.0 and 2.0.

Category	AMI 1.0 (~2023)	AMI 2.0 (2024~)
Aggregator	DCU (firmware update)	SMGW (OS + application program update)
Services	Meter reading, OMS	Meter reading, OMS, smart inverter control, voltage management with ADMS
Security	AMI HE ⟷ DCU (PKI)	AMI HE ⟷ smart meter (PKI)
Device management	SNMP (version 2)	LwM2M
Metering protocol	AMI HE ⟷ DCU (proprietary) DCU ⟷ smart meter (DLMS)	AMI HE ⟷ SMGW (LwM2M) SMGW ⟷ smart meter (DLMS)

**Table 2:** DLMS security (SMGW ⟷ smart meter) comparison.

Parameters	AMI 1.0 (~2023)	AMI 2.0 (2024~)
Mutual authentication	Low-level security with password	High-level security with digital certificate (mechanism ID 7)
Key agreement	-	Static unified model
Encryption	-	Service-specific dedicated ciphering
Digital signing	-	General signing

**Table 3:** OBIS code structure and usage example.

OBIS (Size)	A (1 byte)	B (1 byte)	C (1 byte)	D (1 byte)	E (1 byte)	F (1 byte)
Meaning	Medium	Channel	Quantity	Processing	Classification	Historical
Range	0~15	0~64	0~255	0~255	0~255	0~255
Example (meaning)	1 (electricity)	1 (index)	1 (active power)	8 (integral 1)	2 (rate 2)	255 (present)

**Table 4:** Comparison of resource management features between DLMS and LwM2M protocols.

Parameters	DLMS	LwM2M
Unique ID	Class ID	Object
Data classification	OBIS code	Object instance
Data value	Attribute	Resource

**Table 5:** Example of resource conversion between DLMS and LwM2M protocols.

Load Profile (LP)	DLMS				LwM2M
	METER INDEX	Class ID	OBIS Code	Attribute Index	URI
P (+)	1	3	1.1.1.8.0.255	2	/03/4353/2048/65298
VAR (Q1)	1	3	1.1.5.8.0.255	2	/03/4357/2048/65298
VAR (Q4)	1	3	1.1.8.8.0.255	2	/03/4360/2048/65298
VA (+)	1	3	1.1.9.8.0.255	2	/03/4361/2048/65298
Date & time	1	8	0.0.1.0.0.255	2	/08/01/0/65298
Status	1	1	0.0.97.97.4.255	2	1/97/24836/65298
P (-)	1	3	1.1.2.8.0.255	2	/03/4354/2048/65298
VAR (Q2)	1	3	1.1.6.8.0.255	2	/03/4358/2048/65298
VAR (Q3)	1	3	1.1.7.8.0.255	2	/03/4359/2048/65298
VA (-)	1	3	1.1.10.8.0.255	2	/03/4362/2048/65298

**Table 6:** KEPCO metering profile items used in the analysis.

Load Profile (LP)		Billing Profile (BP)		Maximum Demand Profile (MDP)		Inst. Profile (IP)	
Item	Bytes	Item	Bytes	Item	Bytes	Item	Bytes
P (+)	4	Reading date & time	12	Reading date & time	12	Occurrence date & time	12
VAR (Q1)	4	Meter ID	11	Meter ID	11	P (+)	4
VAR (Q4)	4	P (+)	20 (4 × 5 rates)	P (+)	20 (4 × 5 rates)	Phase angle	4
VA (+)	4	VA (+)	20 (4 × 5 rates)	Occurrence date & time	60 (12 × 5 rates)	Voltage THD	4
Occurrence Date & time	12	VAR (Q1)	20 (4 × 5 rates)	Accumulated P (+)	20 (4 × 5 rates)	Voltage	4
Status	3	VAR (Q4)	20 (4 × 5 rates)	VA (+)	20 (4 × 5 rates)	Current	4
P (-)	4	Power factor	20 (4 × 5 rates)	Occurrence date & time	60 (12 × 5 rates)	Power factor	4
VAR (Q2)	4	-	-	Accumulated VA (+)	20 (4 × 5 rates)	Frequency	4
VAR (Q3)	4	-	-	-	-	Temperature	4
VA (-)	4	-	-	-	-	-	
Subtotal	47	Subtotal	123	Subtotal	223	Subtotal	44

**Table 7:** Analysis of the packet size for three different remote metering approaches.

Parameter			Full DLMS	DLMS to LwM2M (Encapsulation)	DLMS to LwM2M (1:1 Conversion)
LP	Request	Header	6 (DLMS)	8 (CoAP)	8 (CoAP)
	packet size	Body or CoAP option	26	5 (/19/2)	17 (/7/4450/32768/65298)
	(bytes)	Subtotal	32	13	25
	Response	Header	6 (DLMS)	8 (CoAP) + 77 (JSON)	8 (CoAP)
	packet size	Body (encoding)	61 (TLV)	72 (JSON Base64)	47
	(bytes)	Subtotal	67	157	55
BP	Request	Header	6 (DLMS)	8 (CoAP)	8 (CoAP)
	packet size	Body or CoAP option	26	5 (/19/2)	15 (/7/98/257/65298)
	(bytes)	Subtotal	32	13	23
	Response	Header	6 (DLMS)	8 (CoAP) + 77 (JSON)	8 (CoAP)
	packet size	Body (encoding)	154 (TLV)	176 (JSON Base64)	123
	(bytes)	Subtotal	160	261	131
MDP	Request	Header	6 (DLMS)	8 (CoAP)	8 (CoAP)
	packet size	Body or CoAP option	26	5 (/19/2)	19 (/7/4450/32768/65298)
	(bytes)	Subtotal	32	13	27
	Response	Header	6 (DLMS)	8 (CoAP) + 77 (JSON)	8 (CoAP)
	packet size	Body (encoding)	269 (TLV)	308 (JSON Base64)	223
	(bytes)	Subtotal	275	393	231
IP	Request	Header	6 (DLMS)	8 (CoAP)	8 (CoAP)
	packet size	Body or CoAP option	26	5 (/19/2)	19 (/7/4450/32768/65298)
	(bytes)	Subtotal	32	13	27
	Response	Header	6 (DLMS)	8 (CoAP) + 77 (JSON)	8 (CoAP)
	packet size	Body (encoding)	54 (TLV)	68 (JSON Base64)	44
	(bytes)	Subtotal	60	153	52

**Table 8:** Required packet size for security.

Parameters	DLMS Packet Size (bytes)	DTLS Packet Size (bytes)
Digital certificate (X.509-based)	472	472
Overhead for session establishment	1639	2364
Overhead for authenticated encryption	23	25

## Data Availability

Not applicable.

## Abbreviations

The following abbreviations are used in this manuscript:

ADMS	Advanced Distribution Management System
AES-GCM	Advanced Encryption Standard Galois Counter Mode
AMI	Advanced Metering Infrastructure
ANSI	American National Standards Institute
APDU	Application Protocol Data Unit
BP	Billing Profile
CoAP	Constrained Application Protocol
COSEM	Companion Specification for Energy Metering
DCU	Data Concentrator Unit
DLMS	Device Language Message Specification
DTLS	Datagram Transport Layer Security
ECDSA	Elliptic Curve Digital Signature Algorithm
ECDH	Elliptic Curve Diffie-Hellman Algorithm
HTTP	Hypertext Transfer Protocol
ID	Identification
IoT	Internet of Things
IP	Instantaneous Profile
JSON	JavaScript Object Notation
KEPCO	Korea Electric Power Corporation
LP	Load Profile
LTE	Long Term Evolution
LwM2M	Lightweight Machine-to-Machine
MDP	Maximum Demand Profile
MQTT	Message Queuing Telemetry Transport
OBIS	Object Identification System
OMS	Outage Management System
P (+, -)	Power (imported, exported)
PKI	Public Key Infrastructure
PLC	Power Line Communication
REST	Representational State Transfer
SHA	Secure Hash Algorithm
SMGW	Smart Meter Gateway
SNMP	Simple Network Management System
TLV	Type Length Value
THD	Total Harmonic Distortion
URI	Uniform Resource Identifier
VA	Volt-Ampere
VAR	Volt-Ampere Reactive
